# Supplementary material for: Association of MACC1 expression with lymphatic metastasis in colorectal cancer: A nested case-control study
Source: PLoS One. 2021 Aug 3;16(8):e0255489. doi: 10.1371/journal.pone.0255489 (PMC8330891; doi:10.1371/journal.pone.0255489)
Supplement: Attachment — Submitted filename: Response to Reviewers.doc [file pone.0255489.s004.doc]

**Response to Reviewer #1:**

Comments:
[1] Additional information and editing of the content provided in figure legend 1 is strongly recommended. There are two blue lines and information for only one is given and this needs clarification. Next, redundancy in the description on what the x and y coordinates are as it is given for each graph. State it at the very end and just once.

**Response:** Thanks for your kind suggestion. We have modified the figure legend for Figure 1. The explanation of the two blue lines is added, and the redundant part is removed. Thanks!

**Modified contents in Figure legend 1：**

Fig 1. Relationship between MACC1 expression level and lymphatic metastasis. (A) A linear relationship between MACC1 expression level and lymphatic metastasis was observed. The blue circle lines on both sides represent 95% confidence interval and the red dot line represents the fitting curves. (B) A nonlinear relationship between MACC1 expression level and lymphatic metastasis was observed after adjusting all variables. The blue circle lines on both sides represent 95% confidence interval and the red dot line represents the fitting curves. (C) A nonlinear relationship between MACC1 expression level and lymphatic metastasis was observed after adjusting all variables in middle age group. Divide the age into three groups: the red dot line represents the lower age group, the green circle line represents the middle age group, and the blue triangle line represents the higher age group. In this figure, the horizontal ordinate represents the expression level of MACC1, and the longitudinal coordinate represents the probability of lymph node metastasis.
